# 晚期非小细胞肺癌*HER2*基因突变靶向治疗进展

**DOI:** 10.3779/j.issn.1009-3419.2020.101.49

**Published:** 2020-12-20

**Authors:** 令平 孔, 殿胜 钟

**Affiliations:** 300052 天津，天津医科大学总医院肿瘤内科 Department of Medical Oncology, Tianjin Medical University General Hospital, Tianjin 300052, China

**Keywords:** 肺肿瘤, 人表皮生长因子受体-2, 酪氨酸激酶抑制剂, 抗体-药物偶联物, Lung neoplasms, Human epidermal growth factor receptor-2, Tyrosine kinase inhibitor, Antibody-drug conjugate

## Abstract

肺癌是最常见的恶性肿瘤，非小细胞肺癌（non-small cell lung cancer, NSCLC）发生率约占所有肺癌的85%。人表皮生长因子受体2（human epidermal growth factor receptor-2, HER2）是ERBB/HER家族中的一种酪氨酸激酶受体，与表皮生长因子受体（epidermal growth factor receptor, EGFR）等其他家族成员一起激活下游信号传导。*HER2*基因突变与许多上皮细胞癌症的恶化程度密切相关，*HER2*基因突变的肿瘤表现出较强的转移能力和侵润能力，对化疗的敏感性也较差，且易复发。目前肺癌驱动基因靶向治疗进展迅速。作为驱动基因，NSCLC中*HER2*基因突变频率相对于*EGFR*较低，但是其肺癌驱动机制明确且部分靶向治疗有效，将来可能成为新的标准治疗手段。本文重点讨论*HER2*基因突变在NSCLC治疗中的研究进展。

## Abstract (en)

Lung cancer is the most common malignancy tumor. Non-small cell lung cancer (NSCLC) accounts for about 85% of lung cancer. Human epidermal growth factor receptor-2 (HER2) is a tyrosine kinase receptor in ERBB/HER family, which activates downstream signal transduction with other family members such as epidermal growth factor receptor (EGFR). *HER2* gene mutation is closely related to the progression of many epithelial cell cancers. Tumors with high expression of HER2 show strong metastasis and invasion ability, poor sensitivity to chemotherapy, and are prone to relapse. At present, lung cancer driven gene targeted therapy has made rapid progress. Although the frequency of *HER2* gene mutation in NSCLC is lower than that of *EGFR*, its driving mechanism in lung cancer is clear and partial targeted therapy is effective, which may become a new standard treatment in the future. This review focuses on the research progress of *HER2* gene mutation in the treatment of NSCLC.

肺癌是发病率和死亡率增长最快且对人类健康和生命威胁最大的恶性肿瘤^[1, 2]^。非小细胞肺癌（non-small cell lung cancer, NSCLC）发生率约占所有肺癌的85%^[3]^。NSCLC有三种亚型：腺癌、鳞状细胞癌、大细胞癌。近年来，肺腺癌中表皮生长因子受体（epidermal growth factor receptor, *EGFR*）、间变性淋巴瘤激酶（anaplastic lymphoma kinase, *ALK*）等驱动基因的靶向治疗进展迅速^[4, 5]^。对于*EGFR*的突变，三代酪氨酸激酶抑制剂（tyrosine kinase inhibitor, TKI）已被开发，正用于肺癌的治疗^[4]^。与化疗相比，靶向治疗明显延长了无进展生存期（progression free survival, PFS）和总生存期（overall survival, OS）。因此，积极探索肺癌靶向治疗，寻求有效靶点成为目前肺癌治疗的目标之一。

人表皮生长因子受体2（human epidermal growth factor receptor-2, HER2）是ERBB/HER家族中另一种酪氨酸激酶受体，形成异源二聚体，与EGFR等其他家族成员一起激活下游信号传导^[6]^。*HER2*基因突变与许多恶性肿瘤的恶化程度密切相关，HER2高表达的肿瘤表现出强的转移能力和侵润能力，对化疗的敏感性也较差，且易复发^[7]^。在包括乳腺癌、胃癌、肺癌等多种恶性肿瘤中，*HER2*基因突变提示预后不良及生存期短^[8-10]^。在乳腺癌、胃癌、胃食管交界癌中，HER2的靶向治疗可以延长患者生存期，并且已经被作为标准治疗^[11, 12]^。同样，在NSCLC中，*HER2*基因突变的患者生存期较一般患者短，这可能是由于此类患者对化疗药原发耐药^[13]^。

在NSCLC中，*HER2*基因突变主要表现为基因扩增和突变（主要为外显子20插入突变）两种形式，两者都可导致HER2激活。在NSCLC中，*HER2*基因突变的检测方法有多种，但目前尚未建立金标准^[14]^。在目前的实验室检测方法中，*HER2*扩增使用荧光原位杂交方法（fluorescence *in situ* hybridization, FISH）或二代测序（next-generation sequencing, NGS）^[15, 16]^; *HER2*突变可用Sanger测序、扩增阻滞突变系统聚合酶链式反应（amplification refractory mutation system polymerase chain reaction, ARMS-PCR）或者NGS^[15-18]^。*HER2*基因扩增和突变相应的发生频率分别为1%-3%和2%-4%^[15, 19-21]^。HER2的外显子20的插入突变，以p.A775_G776insYVMA多见，还包括p.G776 > VC、p.P780_Y781insGSP、p.V777_G778insCG、p.M774delinsWLV、p.G776 > LC和点突变p.L755S、p.G776C和p.V777L。虽然NSCLC中*HER2*基因突变频率相对于EGFR较低，但是其肺癌驱动机制明确且部分靶向治疗有效，目前临床上已经将其作为一线治疗IV期NSCLC的有效靶点进行筛查。以下就相关治疗药物和临床研究进行综述。

## 1 抗体-药物偶联物（antibody-drug conjugate, ADC）

### 1.1 T-DM1（Trastuzumab Emtasine）

T-DM1是一种半合成药物，即曲妥珠单抗-美坦新偶联物，美坦新为微管抑制剂，药物抗体比（drug antibody ratio, DAR）为3.5。目前有以下三个临床研究。

日本研究者报告了T-DM1单药对复发HER2阳性[免疫组化（immunohistochemistry, IHC）3+，IHC 2+和FISH+或外显子20突变]NSCLC的一项Ⅱ期临床研究，该研究中15例可评估患者的临床特征：中位年龄67岁，HER2状态：IHC 3+：33%;IHC 2+/FISH：20%;突变：A775_G776insYVMA（33%）、P780_Y781insGSP（7%）G776VinsC（7%）。1例部分缓解（partial response, PR），客观缓解率（objective response rate, ORR）为6.7%（90%CI: 0.2%-32.0%）。中位随访时间9.2个月，中位PFS和中位OS分别为2.0个月（90%CI: 1.2-4.0, 95%CI: 1.4-4.0）和10.9个月（90%CI: 2.3-, 95% CI: 4.4-12.0）。3级/4级不良事件包括血小板减少症（40%）和肝毒性（20%），但未见任何治疗相关性死亡。这项研究因为疗效有限而提前终止^[22]^。

美国杜克大学Tom Stinchcombe博士牵头，瑞士、德国、意大利等多个国家研究人员共同开展一项Ⅱ期临床研究^[23]^，共49例转移性或不可切除局部晚期NSCLC患者接受TDM1治疗，其中29例患者IHC 2+，20例患者IHC 3+。结果表明IHC 3+组，ORR为20%，中位生存时间为15.3个月。IHC 2+组ORR为0%（95%CI: 0.0-11.9），中位生存时间为12.2个月。IHC 2+和IHC 3+患者的中位PFS分别为2.6个月和2.7个月。该研究显示TDM1在HER2高表达晚期NSCLC患者中显示出治疗活性，但是HER2免疫组化作为单一参数是不充分的预测标志物。

另外一项来自美国纪念斯隆-凯特林癌症中心的Li博士及其同事联合开展的研究^[24]^，显示了在*HER2*突变的NSCLC患者中，TDM1具有有效性并且安全可耐受。共有18例*HER2*扩增或突变的肺腺癌患者接受治疗，中位年龄为63岁，其中72%为女性，39%为从不吸烟。既往接受的全身性疗法的中位数量为2种。ORR为44%（95%CI: 22%-69%），达到了主要研究终点。在*HER2*外显子20插入、跨膜和细胞外结构域点突变（A775_G776insYVMA, G776delinsVC, V659E, S310F）的患者中可以观察到疾病缓解。毒性主要为1级或2级，包括输液反应、血小板减少和转氨酶升高，未出现降低剂量或与治疗相关的死亡。这是肺癌HER2分子亚群的第一个阳性临床研究，有必要进一步的多中心研究。

### 1.2 DS-8201（trastuzumab deruxtecan, T-DXd）

DS-8201是二代ADC药物，由抗HER2的IgG1单抗通过连接体，与拓扑异构酶I抑制剂Dxd（效能比伊立替康高10倍）组成。DS-8201抗体与药物由可切割的四肽连接体相连，药物抗体比（drug antibody ratio, DAR）高达8。2020年美国临床肿瘤学会（American Society of Clinical Oncology, ASCO）会议上公布了DS-8201（T-DXd）的Ⅱ期临床研究的中期结果，DESTINY-Lung01为一项开放标签、多中心、多队列的Ⅱ期临床研究，此次报道T-DXd在*HER2*突变的晚期NSCLC中的疗效。共入组42例患者，45.2%的患者伴随基线中枢神经系统转移。入组患者中位既往治疗线数为2（范围：1-6）。入组人群中90.5%的患者为伴随*HER2*激酶结构域突变，4.8%的患者为*HER2*胞外结构域突变，余4.8%的患者*HER2*突变特征不详。截至2019年11月，45.2%（19/42）的患者仍在治疗中，终止治疗的患者大多由于疾病进展或不可耐受的不良反应。40例患者可以评估疗效，其中1例完全缓解（complete response, CR）、25例PR、2例疾病稳定（stable disease, SD）、2例疾病进展（progressive disease, PD），ORR为61.9%（95%CI: 45.6%-76.4%），疾病控制率（disease control rate, DCR）为90.5%（95%CI: 77.4%-97.3%）。中位PFS达14.0个月（95%CI: 6.4-14.0），中位缓解持续时间（duration of response, DOR）以及OS尚未达到。3度以上不良反应率为52.4%，严重不良反应率为16.7%。不良反应相关治疗终止率达23.8%，治疗减量率为38.1%，治疗中断率为59.5%。尚无治疗相关死亡病例。DESTINY-Lung01观察到的疗效与安全性与前Ⅰ期研究结果一致^[25]^。

## 2 酪氨酸激酶抑制剂（tyrosine kinase inhibitors, TKIs）

### 2.1 阿法替尼

早在2012年，*Lung Cancer*上首次报道了3例阿法替尼治疗*HER2*外显子20插入突变（分别为p.Tyr772 Ala775dup、p.Gly776Leu、p.Gly778 Pro780dup）的非吸烟肺腺癌患者，在应用阿法替尼2周内，可观察到明显的疾病缓解，但缓解所持续的时间很短，从阿法替尼治疗开始到死亡的时间为12个月-32个月^[26]^。这个报道预示着阿法替尼可能成为*HER2*基因突变NSCLC有效的靶向治疗药物。随后在2013年*Journal of Clinical Oncology*报道，回顾性分析了65例带有*HER2*外显子20插入突变的肺腺癌，其中3例接受阿法替尼单药治疗，均取得疾病缓解，3例应用其他HER2靶向治疗方案的患者均没有取得疾病缓解^[27]^。Costa等^[28]^研究报道了3例阿法替尼脉冲式给药方案（每周一次，280 mg）治疗*HER2*外显子20插入突变的肺腺癌，1例既往未接受过靶向药治疗的患者取得了5个月的部分缓解，另1例患者达到了11个月的病情稳定，还有1例患者对阿法替尼无应答。

美国纽约斯隆凯特琳癌症中心收集了27例接受阿法替尼治疗的晚期*HER2*变异的NSCLC病例，23例疗效可评价，有效率为13%，此外有57%的患者疾病稳定。疗效维持的中位时间为6个月，中位OS为23个月^[29]^。

另外一项前瞻性Ⅱ期临床试验，仅入组了13例患者，DCR为53.8%，但是未观察到客观缓解的患者，因此，该临床试验被提前叫停^[30]^。

阿法替尼虽为EGFR和HER2酪氨酸激酶的强效、不可逆的双重抑制剂，其临床疗效仍不确切。在这个精准治疗时代，寻找更为有效的靶点是提高治疗有效率的重要手段。中山大学附属医院张力教授团队在*The Oncologist*发表一项真实世界的研究^[31]^，此研究中纳入32例多线治疗失败后接受阿法替尼治疗的*HER2*突变肺癌患者，总体ORR和DCR分别为16%和69%。研究者进一步将不同*HER2*突变类型的疗效细分，结果发现：A775_G776insYVMA突变类型的患者阿法替尼的ORR为0%（0/14），DCR为35.7%，而G778_P780dup或G776delinsVC突变类型的患者中阿法替尼的有效率则为40%（4/10），DCR为100%。A775_G776insYVMA突变患者的中位PFS仅为1.2个月，G778_P780dup或G776delinsVC突变患者中位PFS达到了7.6个月（*P*=0.015）。这一数据为阿法替尼在治疗*HER2*突变的NSCLC中筛选出可获益的靶点，更加证实了*HER2*突变型肺癌具有显著的异质性。

### 2.2 达克替尼

2015年，在*Annals of Oncology*上报道一项达克替尼治疗*HER2*突变或扩增肺癌的Ⅱ期临床研究^[32]^，结果显示：30例*HER2*扩增或者*HER2*激活突变的晚期肺癌患者，接受达克替尼治疗，其中26例为*HER2*的外显子20突变患者，3例患者治疗有效，客观有效率仅为12%，而4例*HER2*扩增患者都未达到客观缓解。

### 2.3 波奇替尼

波奇替尼是一个专门针对*EGFR*和*HER2*基因外显子20突变而设计的特异性靶向药。有研究^[33]^表明，波奇替尼可诱导*EGFR*或*HER2*的外显子20插入突变的NSCLC细胞死亡，并且在动物试验中证明波奇替尼可对能成为潜在的EGFR或HER2 20ins治疗药物。波奇替尼Ⅱ期开放性试验在美国安德森癌症中心（NCT03066206）进行。试验队列分为两组（*EGFR* 20ins及*HER2* 20ins+），纳入NSCLC患者（排除T790M+）的治疗线数≥1，包括无症状及稳定的脑转移患者，以便观察波奇替尼对转移的疗效。主要研究终点为ORR，次要终点为PFS、OS、DCR和安全性。在2018年世界肺癌大会上的报道，*HER2*外显子20突变入组了13例患者，12例可评估病例中，ORR达到了50%，中位PFS为5.1个月，有5例仍在接受治疗中。

### 2.4 吡咯替尼

吡咯替尼是小分子不可逆酪氨酸激酶抑制剂（靶点包括EGFR、HER2和HER4），与胞内激酶区ATP结合位点共价结合，全面阻断HER家族同异源二聚体的形成，抑制肿瘤细胞生长并可透过血脑屏障。

在体内外试验中，吡咯替尼具有抑制*HER2*外显子20突变NSCLC细胞活性的作用，吡咯替尼在*HER2*外显子20 A775_G776YVMA插入突变肺腺癌类器官模型和裸鼠PDX模型实验中的抗瘤活性显著优于T-DM1和阿法替尼^[34]^。

在一项Ⅱ期临床试验中，共15例晚期*HER2*突变NSCLC患者接受吡咯替尼治疗，400 mg每天一次，其中10例为*HER2* A 775_G776 YVMA插入突变。ORR为53.3%，中位PFS为6.4个月，中位DOR为7.2个月^[34]^。

今年发表于*Journal of Clinical Oncology*杂志上的一项单臂、开放标签的多中心Ⅱ期临床研究^[35]^，共计86例携带*HER2*突变的患者接受筛查，60例患者入组。41.7%的患者既往接受过至少2种治疗方案，没有患者接受过免疫治疗。患者突变类型如下：外显子20有12个碱基对插入突变（71.3%），G776突变（12%），外显子20有9个碱基对插入突变（8.3%），V777L突变（1.7%），L755P突变（6.7%）。中位随访时间为11.7个月，81.7%的患者停止治疗。研究者评估的ORR为31.7%，均为PR，反应持续时间为7.0个月，PFS为6.9个月（图3）。中位OS为14.4个月。3级治疗相关不良事件发生率为26.7%，主要不良事件为腹泻（20%），1例患者出现4级γ-谷氨酰转移酶升高。该研究结果初步验证了吡咯替尼作为单药治疗*HER2*外显子20突变NSCLC患者的疗效与安全性。

### 2.5 TAK-788（AP32788）

TAK-788是一种具有口服活性的靶向*EGFR*和*HER2*突变的小分子抑制剂。2016年美国癌症研究学会（American Association for Cancer Research, AACR）首次基础研究报告了TAK-788对14个*EGFR*突变株和6个*HER2*突变株均有效^[36]^。NCT02716116是一项Ⅰ期/2期开放标签多中心研究，用来评价EGFR/HER2抑制剂TAK-788治疗的安全性、药代动力学和抗肿瘤活性以及在非NSCLC的*EGFR*或*HER2*突变实体瘤患者中的抗肿瘤活性。2018年ASCO上，研究者报告了TAK-788研究剂量递增阶段结果，纳入患者均为*EGFR*外显子20插入或*HER2*外显子20突变患者。截至2017年9月8日，共纳入34例。在14例可评价的患者中，3例PR，6例SD，5例PD; 研究汇总显示所有PR或SD的患者都有*EGFR*外显子20插入突变。因此，后续的扩增研究对*EGFR*外显子20插入突变进行分析研究，2019年报道了TAK-788对于NSCLC *EGFR*外显子20插入突变的治疗效果，试验结果表明，*EGFR*外显子20插入突变患者的ORR为43%，PR为43%，SD为43%，DCR为86%，中位PFS为7.3个月^[37]^。*HER2*突变治疗效果尚没有报道，继续关注该临床研究结果。

## 3 总结

根据以上临床研究的结果，*HER2*突变在晚期NSCLC尚不能作为一线治疗，但是在DESTINY-Lung01研究中，二代ADC药物DS-8201取得了惊艳的成绩，ORR为61.9%，DCR为90.5%，中位PFS达14.0个月，该研究结果达到了*EGFR*常见突变位点靶向治疗疗效。同样，吡咯替尼治疗*HER2*突变患者的ORR为31.7%，PFS为6.9个月。以上两项研究有望成为*HER2*突变的治疗新选择。但上述研究均为Ⅱ期小样本、后线临床研究，今后仍需扩大样本的研究，为抗*HER2*突变的NSCLC靶向治疗提供有力证据。

此外，上述临床研究中治疗有效的患者*HER2*改变形式均为突变，目前尚未发现*HER2*扩增在NSCLC中有效的靶向药物。并且在*HER2*突变的患者中，不同的突变位点及类型，对靶向治疗的反应亦不相同，张力教授团队关于阿法替尼真实世界的研究，充分表明*HER2*突变型NSCLC具有显著的异质性。突变位点的精准筛选有望大幅度提升HER2抑制剂在人群中的有效率; 同时高质量的基因检测是精准筛选患者人群、继而决定治疗策略的基础。

综上所述，*HER2*突变可能为NSCLC的重要驱动基因，类似于*EGFR*、*ALK*等。期待将来Ⅱ期临床试验扩大样本后取得好的结果，尤其是二代ADC药物DS-8201。同时，从精准治疗的角度出发，寻找不同HER2抑制剂有效的*HER2*突变类型，从而达到最佳的治疗效果。
